# Recurrent Activity in Higher Order, Modality Non-Specific Brain Regions: A Granger Causality Analysis of Autobiographic Memory Retrieval

**DOI:** 10.1371/journal.pone.0022286

**Published:** 2011-07-27

**Authors:** Hans C. Lou, Morten Joensson, Katja Biermann-Ruben, Alfons Schnitzler, Leif Østergaard, Troels W. Kjaer, Joachim Gross

**Affiliations:** 1 Center of Functionally Integrative Neuroscience, Aarhus University, Aarhus, Denmark; 2 Department of Neurology, Heinrich Heine University, Düsseldorf, Germany; 3 Department of Clinical Neurophysiology, Rigshospitalet, University of Copenhagen, Copenhagen, Denmark; 4 Department of Psychology, University of Glasgow, Glasgow, United Kingdom; French National Centre for Scientific Research, France

## Abstract

It has been proposed that the workings of the brain are mainly intrinsically generated recurrent neuronal activity, with sensory inputs as modifiers of such activity in both sensory and higher order modality non-specific regions. This is supported by the demonstration of recurrent neuronal activity in the visual system as a response to visual stimulation. In contrast recurrent activity has never been demonstrated before in higher order modality non-specific regions. Using magneto-encephalography and Granger causality analysis, we tested in a paralimbic network the hypothesis that stimulation may enhance causal recurrent interaction between higher-order, modality non-specific regions. The network includes anterior cingulate/medial prefrontal and posterior cingulate/medial parietal cortices together with pulvinar thalami, a network known to be effective in autobiographic memory retrieval and self-awareness. Autobiographic memory retrieval of previous personal judgments of visually presented words was used as stimuli. It is demonstrated that the prestimulus condition is characterized by causal, recurrent oscillations which are maximal in the lower gamma range. When retrieving previous judgments of visually presented adjectives, this activity is dramatically increased during the stimulus task as ascertained by Granger causality analysis. Our results confirm the hypothesis that stimulation may enhance causal interaction between higher order, modality non-specific brain regions, exemplified in a network of autobiographical memory retrieval.

## Introduction

Bi-directional, or re-entrant, re-activation of regions in cortico-thalamic networks has been proposed to account for sufficient strength and duration of their activity to induce conscious visual perception [1], [2]. This hypothesis is supported by the finding that visual stimulation may elicit recurrent, bidirectional, and, presumably, reciprocal re-enforcing neural activity in the visual system [3]–[5].

**Figure 1:**
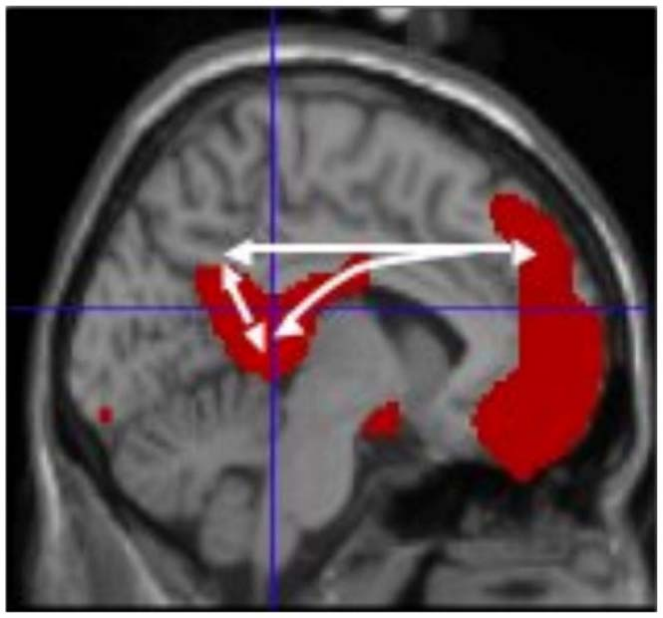
A paralimbic network of self-awareness. The network was identified in a previous PET study of hemodynamic paralimbic interactions elicited by self-awareness [12]). Talairach coordinates of anterior cingulate/medial prefrontal, posterior cingulate/medial parietal, and pulvinar thalami were measured for identification of targets for the MEG analysis used in the present study (0,59,40; 0,−50,28; and 0,−38,8, respectively).

In higher-order, modality non-specific brain regions, recurrent activity has never been recorded in humans before. The aim of the present study is to test the hypothesis that stimulation may enhance causal recurrent interaction between higher order, modality non-specific brain regions. As stimuli we used autobiographic memory retrieval of judgment of visual words, and examined neuronal activity in a paralimbic network which participates in autobiographic memory retrieval [6], [7] and self-reference [8], [9] (Fig. 1). The network includes medial prefrontal/anterior cingulate, medial parietal/posterior cingulate cortical regions, together with the pulvinar thalami.

MEG [10], [11] was used to target the midline between right and left at the following locations: anterior cingulate/medial prefrontal cortex (AC) (Talairach coordinates 0, 59, 40); posterior cingulate/medial parietal cortex (PC) (0, −50, 28); and pulvinar thalami (Thal) (0, −38, 8).

## Methods

### Participants

Recordings were obtained from 12 healthy, right-handed, gender-balanced matched native German speakers (mean age 30 years, SD 5 years) with the local ethics committee's approval. All subjects gave their informed consent in writing.

### Experimental procedure

**Figure 2:**
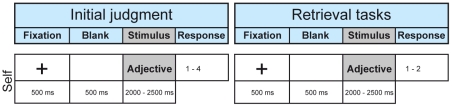
Time-lines of the experiments. Three different blocks of 40 different, randomly selected, common adjectives were used. Initially a personal judgment was recorded on a four-point scale of how well each adjective fitted him or herself. About 4 min after personal judgment of each adjective, the same adjectives were shown again in a different order for autobiographical memory retrieval. Retrieval response was made on a two-point scale (rather fitting or not fitting). Data collection for the present study was limited to the retrieval process. The order of the blocks was balanced to obtain an even distribution.

A series of 120 different German adjectives was split into three blocks of 40 words sequentially presented on a back-projection screen at a distance of 1.2 m from the participant [12]. Each word presentation was preceded by a 500 ms long presentation of a fixation cross and a 500 ms presentation of a blank screen. Participants were trained to avoid eye movements. The presentation time for each word varied randomly in the interval between 2000 and 2500 ms in order to make it less straightforward for the participant to know when to respond. Immediately after word disappearance, a response screen was presented consisting of the four choices for the judgment about reference to Self (−−− (does not match at all), − (matches poorly), + (matches reasonably well), +++ (matches absolutely)). The subject was allowed to take as long as 5 s for the decision. Then the next word was presented for encoding. When all 40 words in a block had been judged, the same adjectives were presented in random order and the previous judgments had to be retrieved. The participants were required to respond as to whether the adjective had previously been judged to be rather fitting or not to him or her-self on a two-point scale. While we wished to use a four-point scale for encoding to ensure sufficient effort to be applied, retrieval was limited to two options to make any automatic motor response unlikely. Each response elicited immediate presentation of the next adjective.The interval between judgment and retrieval of judgment for each adjective varied and was 4 min on average. MEG data collection for the present study was limited to the initial prestimulus condition and to the retrieval process of judgment of one-self [12] (time line in Fig. 2). Participants had been trained to avoid eye movements. This was successful as ascertained by visual inspection for eye movement artifacts on the MEG tracing.

### Magnetoencephalography

High-resolution T1-weighted magnetic resonance images were obtained from each participant for anatomic co-registration. The original Talairach coordinates were inversely transformed into individual coordinate systems for computation of local activations in each participant. Neural activity was recorded with a Neuromag (Helsinki) 122 whole-scalp neuromagnetometer in a magnetically shielded room. MEG signals were recorded with a pass-band of 0.03–333 Hz and digitized with 1,000 Hz. Time series of activations were computed using a LCMV beamformer [13]. The beamformer analysis was computed with suitable coefficients for a linear combination of channel recordings. Computation of coefficients tries to achieve an optimal focal spatial pass-band filter that passes activity from the respective region of interest with unit gain while simultaneously suppressing activity from other areas [14]. Conservatively estimated, this method has a spatial resolution of at least 10 mm for the cortical regions, and 20 mm for thalamus [10], [11].

### Data analysis

The Granger causality test was used for analysis of MEG data [15]. This approach has recently been introduced in consciousness research to identify bi-directional, mainly posterior-anterior connectivity in the brain [16]. Bi-directional activity describes reciprocal interaction between one neuron or neuronal assembly (I) with another (II). It is presumed that (I) activates (II), which in turn re-activates (I), etc., to increase signal intensity and duration [17], [18]. To determine whether signal A “Granger causes” B, a multivariate autoregressive-model (AR) for signal B is created mathematically. In this model previous values of both signal A and B as well as other measured signals are included, and a residual (E) accounts for the prediction error between the model and actual data. If the coefficient **k** for signal A is different from zero, this indicates that the prediction of B is improved by inclusion of A. Formally this is tested using an F-test with the null-hypothesis **k** = 0 and the calculated test-variable is used as input in the ANOVA-calculations presented in this article. True causality can, however, only be established if it can be ruled out that their interaction depends on a third, external influence [19]. We have ascertained this in a previous study, confirmed and extended in another laboratory [12], [20], by using TMS targeting the medial parietal and medial pre-frontal regions of the circuitry. Reversible disruption of normal activity of each of these regions reduced self-reference selectively and temporarily, showing that both regions are effective in self-reference. A demonstration of bi-directional Granger causality in their interaction would therefore reveal causal recurrent reactivation in the paralimbic network for self-reference. [21].

The test variables were computed on single trials in 100 ms time windows that covered the time range between −1 s and +1 s with respect to stimulus onset. Time series were computed for each single trial, for each region of interest and for each individual participant. These time series were subjected to directionality analysis using Granger causality as implemented in the Causal Connectivity Analysis Toolbox [15]. We calculated 95% confidence intervals of all F-values from Granger causality analysis. This made it possible to ascertain bi-directionality between each set of two regions.

## Results

### Behavioral data

The mean correct episodic retrieval rate was 94.9% (range 88.3–100%). The high rate indicates that the experimental procedure was adequate and demonstrated that the participants did their best to comply with the requirements of the tasks. (Primary data have been presented previously in another context [22]).

### MEG data

**Figure 3:**
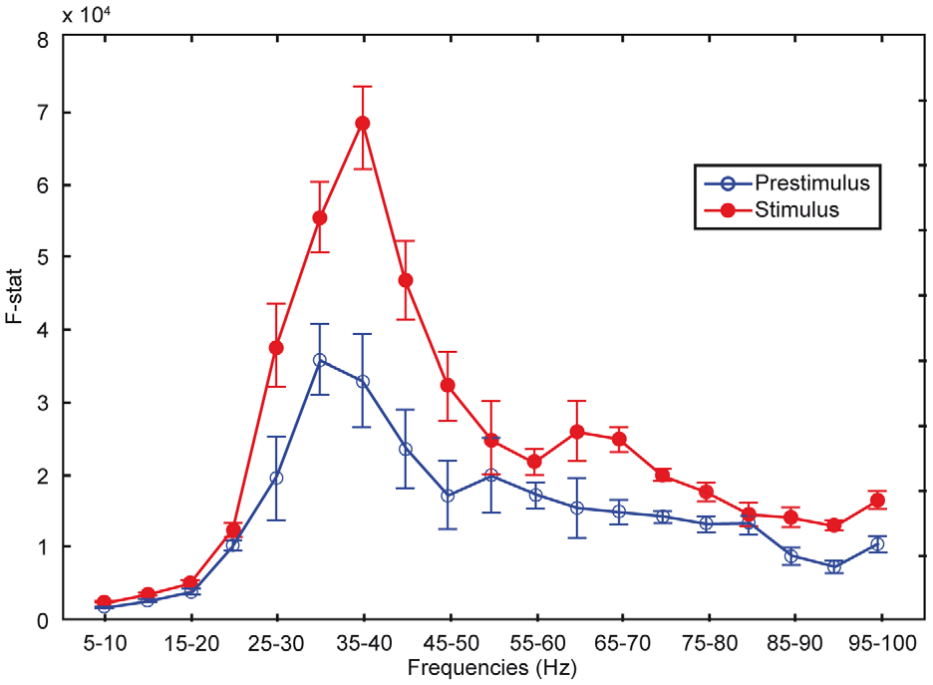
Paralimbic causal information flow during 900 ms prestimulus and 900 ms stimulus conditions vs. 5 Hz frequency bands. Granger causality is seen in both the prestimulus state and during retrieval of self-judgment. Granger causality was maximal in the lower gamma band 30–45 Hz range (bars: standard errors of mean), and larger in the autobiographic memory retrieval condition than in the prestimulus condition (p<0.0001, ANOVA).

Granger causality was determined during 1 s in the prestimulus condition and during 1 s in the stimulus condition. Three-factorial ANOVA was computed for the prestimulus condition and the first second of the stimulus condition. Total interaction between the three paralimbic structures was calculated with the factors condition (prestimulus and stimulus), and 5 Hz frequency bands (5–10,10–15, … 95–100 Hz). ANOVA revealed a highly significant main effect of condition. with maximum in the lower gamma range at 30–45 Hz (p<0.0001, Fig. 3).

**Figure 4:**
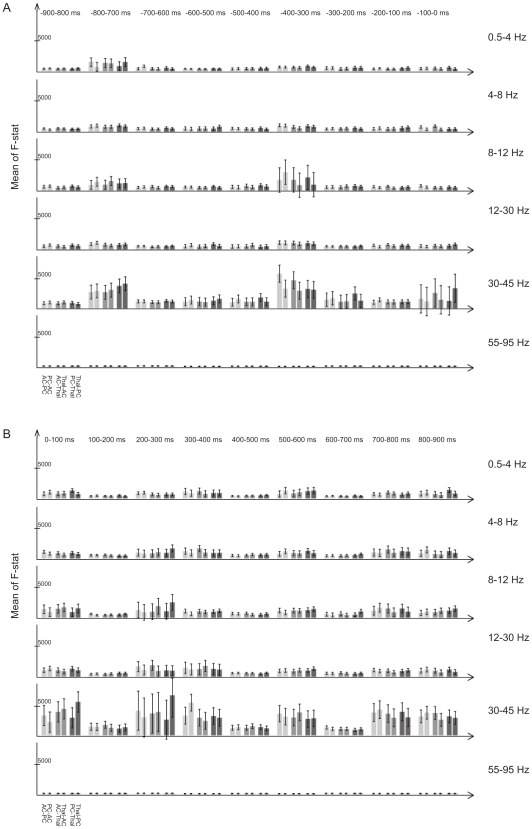
Bi-directional paralimbic interregional activity in the prestimulus condition (A) and in the stimulus condition (B). Granger causality is bi-directional and approximately symmetrical between regions in almost all 100 ms epochs with few exceptions, independent of frequency band, and in both conditions, although intensified in the lower gamma range and during autobiographical memory retrieval. Bars: 95% confidence limits.

Figure 4 shows an analysis of causal paralimbic interactions in 100 ms epochs separately for the last 900 ms during pre-stimulus condition (a), and for the first 900 ms in the stimulus condition during the self-awareness task (b). With few exceptions causal interactions were bi-directional between regions, confirming the hypothesis of bi-directionality of causal influences in the paralimbic circuitry. The epochs are organized in a cyclic pattern with low causality (below 2000F) alternating with epochs with higher causality (up to 7000F) in the lower gamma range (30–45 Hz), where maximal activity was seen. The high causality epochs in this frequency range were fewer before stimulation onset than after (2–3 per second vs. 6–7 per second) (Fig. 4), in accordance with the effect of condition (Fig. 3).

## Discussion

The study has demonstrated a pattern of causal recurrent oscillations mainly in the lower gamma range (30–45 Hz) in a paralimbic cortico-thalamic network in the prestimulus state. During retrieval of judgment of visually presented words in an autobiographic memory retrieval task, the 30–45 Hz oscillations were strongly enhanced. These results confirm and extend the hypothesis that stimulation may enhance causal recurrent interaction between higher order, modality non-specific brain regions.

Two opposing views have prevailed in the discussion on whether the neural activity in the brain needs to be triggered by sensory stimulation, or rather is an internal state of the brain. In the former view the nervous system is organized as a set of complex neuronal connectivity patterns triggered into action by the outside world [23]; accordingly, behavior is fundamentally the resultant of the external world. In contrast, the latter line of thought sees the workings of the brain as mainly intrinsically generated neuronal activity, with sensory inputs acting as modifiers of such intrinsic activity [24], [25]. According to this view, emergence of conscious experience is achieved through oscillatory binding of disparate regions in the brain. Although the design of the present study does not aim at separating preconscious processing from conscious processing, the present results are in accordance with the latter concept. This interpretation is supported by experimental studies of absence seizures. Absence seizures of childhood offers nature's cleanest example of reversible and isolated impairment of conscious experience. The seizures are non-convulsive, generalized brief decreases of consciousness interrupting normal behaviour. Sometimes minimal facial jerks and eye blinks are present. EEG is characteristic, showing well-defined 3 Hz spike-wave discharges. In a genetic rodent model of the disorder, Granger analysis has revealed that abnormally increased bi-directional cortico-thalamic activity was invariably linked to absences [26]. This correlation is consistent with our finding of causality of bi-directional cortico-cortical and cortico-thalamic re-activation in higher order regions responsible for autobiographic memory retrieval and self-reference. In humans, disturbance of the cortico-thalamic interaction has been identified in a wide range of human clinical conditions with dysfunctional self-reference and conscious experience like epilepsy [27], Alzheimer's disease [28], ADHD [29] and, most severely, the vegetative state [30].

Several investigators have proposed that recursive activity in cortico-thalamic interaction may bootstrap neuronal processing in order to elicit conscious experience [4], [31]–[34]. Such a mechanism is supported by anatomical studies showing abundant recurrent synapses in the cortex [32] and by physiologic studies of the visual system [4], [31], [33], [34], although challenged recently by modeling studies [35]. The most convincing in vitro studies in support of recurrent amplification of cortico-thalamic interaction are electrophysiological studies of isolated cortico-thalamic slices [31]. This study combines precise anatomical data and physiological data in an isolated preparation devoid of external influences. Our finding that recurrent activity is a feature of cortico-cortical and cortico-thalamic interaction in the paralimbic network in vivo is further support for this theory. This does not mean that recurrent activity would have to be specifically related to autobiographic retrieval. On the contrary, it seems likely from anatomical and functional studies in vitro and in other species cited above that causal recurrent activity is a fundamental characteristics of cortico-cortical interaction for any function. Further in vivo studies will have to clarify this issue.

The re-entrant connections imply feedback to modify the very stimulation to which they are a response. Such recurrence of activity therefore provides a basis for an extended “subjective present”, as opposed to the instantaneous “physical present” [36]. This extension is thought to occur by incorporating elements from the immediate past and future, as formulated by Blaise Pascal: “We never keep to the present. We anticipate the future as we find it too slow in coming and we are trying to hurry it up, or we recall the past as if to stay its too rapid flight [36]”.

### Limitations

The study does not tell us whether the recurrent activity encountered in the prestimulus condition and amplified with processing complex visual stimuli is a general phenomenon in cortico-cortical and cortico-thalamic processing. There is no evidence to suggest that the recurrent activity demonstrated here should not occur elsewhere in the brain and in any cortico-cortical or cortico-cortical interaction. This question invites to further studies.

## References

[1] Tononi G, Koch C (2008). The neural correlates of consciousness, An update.. Ann NY Acad Sci.

[2] Edelman GM (1989). The remembered present: A biological theory of consciousness.

[3] Pasqual-Leone A, Walsh V (2001). Fast backprojections from motion to the primary visual area are necessary for visual awareness.. Science.

[4] Scholte HS, Jolij J, Fahrenfort JJ, Lamme VA (2008). Feedforward and recurrent processing in scene segmentation: electroencephalography and functional magnetic resonance imaging.. J Cogn Neurosci.

[5] Camprodon JA, Zohary E, Brodbeck V, Pasqual-Leone A (2010). Two phases of V1 for visual recognition of natural images.. J Cogn Neurosci.

[6] Maguire EA (2001). Neuroimaging studies of autobiographical event memory.. Philos Trans R Soc Lond B Biol Sci.

[7] Daselaar SM, Rice HJ, Greenberg DL, Cabeza R, LaBar KS (2008). The spatiotemporal dynamics of autobiographical memory: neural correlates of recall, emotional intensity, and reliving.. Cereb Cortex.

[8] Kjaer TW, Nowak M, Lou HC (2002). Reflective self-awareness and conscious states: PET evidence for a common midline parietofrontal core.. Neuroimage.

[9] Northoff G, Bernpohl F (2004). Cortical midline structures and the self.. Trends Cogn Sci.

[10] Gross J, Kujala J, Hamalainen M, Timmermann L, Schnitzler A (2001). Dynamic imaging of coherent sources: Studying neural interactions in the human brain.. Proc Natl Acad Sci USA.

[11] Gross J, Timmermann L, Kujala J, Salmelin R, Schnitzler A (2003). Properties of MEG tomographic maps obtained with spatial filtering.. NeuroImage.

[12] Lou HC, Luber B, Crupain M, Keenan JP, Nowak M (2004). Parietal cortex and representation of the mental self.. Proc Natl Acad Sci USA.

[13] Ahonen A (1993). 122-channel squid instrument for investigating the magnetic signals from the human brain.. Physica Scripta.

[14] van Veen B, van Drongelen W, Yuchtman M, Suzuki A (1997). Localization of brain electrical activity via linearly constrained minimum variance spatial filtering.. IEEE Trans Biomed Eng.

[15] Seth AK (2005). Causal connectivity of evolved neural networks during behavior.. Network.

[16] Gaillard R, Gaillard R, Dehaene S, Adam C, Clémenceau S (2009). Converging intracranial markers of conscious access.. Plos Biology.

[17] Libet B (1982). Brain stimulation in the study of neuronal functions for consciousness.. Hum Neurobiol.

[18] Libet B, Pearl DK, Morledge DE, Gleason CA, Hosobuchi Y (1991). Control of the transition from sensory detection to sensory awareness In man by the duration of a thalamic stimulus. The cerebral “time-on” factor.. Brain.

[19] Hacker RS, Hatemi-JA (2006). Test for causality between integrated variables using asymptotic band bootstrap distributions: theory and applications.. Applied Economics.

[20] Kwan VS, Barrios V, Ganis G, Gorman J, Lange C (2007). Assessing the neural correlates of self-enhancement bias: a transcranial magnetic stimulation study.. Exp Brain Res.

[21] Gallagher S (2000). Philosophical conceptions of the self: Implications for cognitive science.. Trends Cogn Sci.

[22] Lou HC, Gross J, Biermann-Ruben K, Kjaer TW, Schnitzler A (2010). Coherence in consciousness: Paralimbic gamma synchrony of self-reference links conscious experiences.. Hum Brain Mapp.

[23] James WT (1890). Principles of Psychology.

[24] Llinas R, Paré D (1991). Of dreaming and wakefulness.. Neuroscience.

[25] Mogilner A, Llinás R (1991). Magnetic field tomography of coherent thalamocortical 40 Hz oscillations in humans.. Proc Natl Acad Sci USA.

[26] Sitnikova E, Dikanev T, Smirnov D, Bezruchko B, van Luitelaar G (2008). Granger causality. Cortico-thalamic interdependencies during absence seizures in WAG/Rij rats.. J Neurosci Methods.

[27] Chavez M, Martinerie J, Le van Quyen M (2003). Statistical assessment of non-linear causality: application to epileptic EEG signals.. Neurosci Methods.

[28] Dauwels J, Vialatte F, Latchoumane C, Jeong J, Cichocki A (2009). An EEG synchrony analysis for early diagnosis of Alzheimer's disease: A study with several synchrony measures and EEG data sets.. Conf Proc IEEE Eng Med Biol Soc.

[29] Castellanos FX, Margulies DS, Kelly C, Uddin LQ, Ghaffari M (2008). Cingulate-precuneus interactions: a new locus of dysfunction in adult attention-deficit/hyperactivity disorder.. Biol Psychiatr.

[30] Laureys S (2004). Functional imaging in the vegetative state.. NeuroRehabilitation.

[31] Llinás R, Ribary U, Contreras D, Pedroarena C (1998). The neuronal basis for consciousness.. Philos Trans R Soc Lond B Biol Sci.

[32] Felleman DJ, van Essen DC (1991). Distributed hierarchial processing in the primate cerebral cortex.. Cerebral Cortex.

[33] Lamme VA, Roelfsema PR (2000). The distinct modes of vision offered by feedforward and recurrent processing.. Trends Neurosci.

[34] Rolls ET, Tovée MJ, Panzeri S (1999). The neurophysiology of backward visual masking: information analysis.. J Cogn Neurosci.

[35] Goldman MS (2009). Memory without feedback in a neural network.. Neuron.

[36] Pascal B (1669). Pensées (transl. A.J. Krailsheimer)..

